# Advancing CEA quantification: Designing a sensitive electrochemical immunosenor using MWCNT/Ni(OH)_2_ nanocomposite

**DOI:** 10.1016/j.heliyon.2024.e29768

**Published:** 2024-04-17

**Authors:** Ali Shamsazar, Mahsa Soheili Moghaddam, Asadollah Asadi, Majid Mahdavi

**Affiliations:** a Department of Biology, Faculty of Science, University of Mohaghegh Ardabili, Ardabil, Iran; b Department of Internal Medicine, Faculty of Medicine, Ardabil University of Medical Sciences, Ardabil, Iran; c Institute of Biochemistry and Biophysics, University of Tehran, Tehran, Iran

**Keywords:** Cancer, CEA, Biomarker, Immunosensor, Ni(OH)_2_, MWCNT

## Abstract

An ultra-sensitive immunosensor was designed for the accurate determination of Carcinoembryonic Antigen (CEA). To enhance the performance of immunosensor, an MWCNT/Ni(OH)_2_ nanocomposite was utilized as the electrochemical interface and modifier of the electrode surface. The simple preparation procedures for MWCNT/Ni(OH)_2_ composite were provided. Its characteristics and properties were investigated by HRTEM, FESEM, XRD, and FTIR techniques. Leveraging the unique electrochemical characteristics shown by the MWCNT/Ni(OH)_2_ nanocomposite and its correlation with CEA, high accuracy in CEA detection was achieved. Experimental findings provide evidence that the proposed immunosensor has the ability to detect CEA in laboratory samples. This research contributes towards achieving precise and rapid CEA detection in cancer diagnosis and prognosis. Across a wide concentration range of CEA, the designed immunosensor demonstrated a linear response from 0.0001 ng/mL to 2 ng/mL, and its limit of detection (LOD) was just 0.076 pg/mL. To evaluate the practical applicability of the electrochemical immunosensor, blood serum samples were examined, revealing the immunosensor's remarkable specificity and longevity. Its high accuracy and stability make it a valuable tool in clinical settings and biomedical research, paving the way for improved cancer management and patient outcomes.

## 1 Introduction

Carcinoembryonic antigen (CEA) with a molecular weight of about 170 kDa is considered a monomeric glycoprotein biomarker for all types of colon, lung, and breast cancers [1,2]. For this reason, knowing the level of CEA concentration is very important to diagnose these diseases, monitor affected patients, and provide effective treatments [3]. On an important note, it can be said that knowing the exact level of this biomarker in body fluids during the chemotherapy of a patient with colon cancer can help the treating group to continue the treatment path taken [4]. It is also important to note that the CEA test is not a definitive test for colon cancer screening, but rather a tool that can be used to monitor disease progression and treatment effectiveness. Normal levels of CEA in the blood can vary depending on the laboratory and the age and gender of the individual, but in general, CEA levels are considered normal if they are less than 2.5–5 ng/mL in non-smokers [5]. There are many other factors and some people with colon cancer may have normal CEA levels and some people without colon cancer may have elevated CEA levels due to other conditions such as smoking or inflammation. The most common method to detect the amount of CEA in the blood is to use the immunoassay technique. This involves the use of antibodies specific to CEA, which are used to identify and measure the amount of protein in a blood sample [6]. Considering the physical condition of some cancer patients in whom there is a need to measure this protein tumor marker and the inability of some of them to tolerate other diagnostic methods offered in clinics, the need and development of an efficient method to detect the concentration level of CEA with high sensitivity and simple operating methods are essential [[7], [8], [9]]. This is to avoid the invasive, complex, and expensive methods for monitoring patients with colon cancer such as biopsy and CT scan [10,11]. Non-enzymatic electrochemical immunosensors based on the interaction of antibodies with antigens as target substances to be identified have recently attracted the attention of researchers due to their rapid response times, attributes of simplicity in operation, excellent sensitivity, and remarkable reliability [12,13]. At higher accuracy levels, electrochemical immunosensors are a promising option for getting beyond these restrictions because of their inherent qualities of simplicity, increased sensitivity, non-invasiveness, quick response, and affordability [14,15]. To provide the best performance of immunosensors, the characteristics of the electrode surface should be suitable for the tight and regular binding of antibodies as receptors for target antigens [16,17]. Various nanomaterials are used to improve the electrochemical activity of the immunosensors surface, such as nano metals, nano metal oxides, composites of nano carbons, and quantum dots in surface modification [18,19]. Carbon nanomaterials as one of the materials that are attached to active functional groups as suitable modifiers in biosensors have been extensively studied by researchers. Graphene and carbon nanotubes (CNTs) can be mentioned as such nanomaterials [20,21]. Multi-walled carbon nanotubes (MWCNTs) have been widely utilized owing to their excellent conductivity, specific surface area, and strong chemical stability. These properties have the potential to significantly enhance the electrochemical performance of biosensors [22]. These materials in the structure of immunosensors, because of their physical properties, when used alone cannot show all these listed characteristics in the measurement environments of target analytes in blood serum samples [23]. Therefore, a complex of these CNTs along with other nanomaterials such as metal oxide nanoparticles can help to improve the inherent characteristics of both materials [24,25]. As reported in previous works, Brince Paul K et al. (2017) designed a biosensor for malaria biomarker detection by combining MWCNTs with ZnO nanofiber [26]. In 2021, Ghazala Ashraf et al. prepared a biosensor for serotonin detection using CuO nanoparticles arranged on CNTs, which showed a detection limit of 3 nM [27]. In 2023, Luyao Li et al. prepared a Field-Effect Transistor (FET) sensor for CEA determination based on a CNT film combined with a yttrium oxide Y_2_O_3_ dielectric layer, which could detect CEA in a broad range of 1 fg/mL to 1 ng/mL, and measure with good accuracy and high sensitivity [28].

Electrodes modified with metal hydroxide nanoparticles in combination with MWCNTs have a larger active surface to stabilize biological elements and have higher conductivity for electron exchange reactions on the surface.

In this work, to increase the active surface of the electrode and create a suitable condition for the attachment of the antibody-antigen sandwich system, COOH-MWCNT/Ni(OH)_2_ nanocomposite was used. This nanocomposite was adopted for the first time to prepare an electrochemical immunosensor in the diagnosis of CEA. Innovatively, to save production costs and provide better performance, the use of enzymes in the structure of the immunosensor was eliminated. Herein, instead of the Horseradish peroxidase (HRP) enzyme, the secondary monoclonal Anti-CEA antibody (Ab_2_) conjugated with Fe_3_O_4_ nanoparticles was used as a signal probe. These Fe_3_O_4_ nanoparticles have the catalytic properties of hydrogen peroxide (H_2_O_2_) [29] that can replace the HRP enzyme. They can also induce the magnetic effect on their side structures and prevent the accumulation of anti-CEA antibodies on each other when binding to fixed CEA [30] on the electrode. This also causes the regular arrangement of Ab_2_ and their uniform binding to CEA antigens. The prepared electrochemical immunosensor had reasonable stability, high selectivity and sensitivity, a wide linear range of 0.0001 ng/mL to 2 ng/mL, and a low limit of detection (LOD) of 0.076 pg/mL for CEA detection.

## 2 Materials and methods

### 2.1 Reagents

Distilled water was utilized in the preparation of all solutions. Dextran, K_3_[Fe(CN)_6_], K_4_[Fe(CN)_6_], K_2_HPO_4_, KH_2_PO_4_, NaOH, KCl, and Ni(NO_3_)_2_.6H_2_O were purchased from Sigma-Aldrich. Fe_3_O_4_ nanoparticles salts, urea, and ethanol were stocked in the laboratory. MWCNTs functionalized with carboxyl groups were purchased from US Nano. Monoclonal anti-CEA antibodies were purchased from Arigo Biolaboratories (Hsinchu, Taiwan) and CEA was purchased from Santa Cruz Biotechnology.

### 2.2 Apparatus

Analysis of nanomaterial structures was done with field emission scanning electron microscopy (FESEM) and energy dispersive X-ray (EDX) techniques with a FESEM, TESCAN MIRA3 microscope. A high-resolution transmission electron microscope (HRTEM) with the Zeiss Libra model was used to examine the morphology of nanocomposite and nanoparticles. Further analyses were conducted on the nanomaterials used in the preparation of the immunosensor with XRD (X-pert pro analytical model) and FTIR (Thermo-Avatar spectrometer). Electrochemical experiments were performed by the conventional three-electrode system.

### 2.3 Preparation of Fe_3_O_4_ nanoparticle attached to the Ab_2_

Fe_3_O_4_ nanoparticles (with enzyme-like function and peroxidation property) conjugated with an anti-CEA antibody became a substitute for an enzyme such as HRP, which is usually used as a signal label attached to antibodies [31]. This newly designed probe was used in the detection of the CEA antigen. To prepare the Ab_2_ conjugated with Fe_3_O_4_ nanoparticles as a tracer antibody, 0.5 g of Fe_3_O_4_ nanoparticles prepared by our previous method were weighed [32]. After dispersing the Fe_3_O_4_ nanoparticles in 20 mL of double distilled water for 15 min, 5 mL of dissolved dextran was added to the Fe_3_O_4_ nanoparticles, and the resulting solution was placed on a stirrer for 1 h. Dextran coated the magnet nanoparticles. Next, 3 mL of NaIO_4_ was added to the solution to create aldehyde groups on dextran to react with surface active groups of Ab_2_. The solution was stirred for 30 min. Subsequently, to allow the interaction between the amino groups of the antibody and the aldehyde group on dextran that covers the Fe_3_O_4_, 10 μL of Ab_2_ (1 mg/mL) was introduced into the solution. In this way, the signaling probe was formed. 5 % BSA was used to block unreacted aldehyde groups on dextran, and additional Ab_2_s that could not bond with aldehyde groups on Fe_3_O_4_ nanoparticles were removed with 0.1 M PBS (pH = 7.4) [33].

### 2.4 Decoration of Ni(OH)_2_ nanoparticles on MWCNTs

Ni(OH)_2_ nanoparticles were synthesized by the coprecipitation method. In this chemical technique, a solution of 0.5 g of Ni(NO_3_)_2_.6H_2_O and 25 mL of distilled water was prepared, Afterwards, while continuously stirring, 10 mL of a 0.1 M NaOH was dropped into the mixture. Then, the precipitate obtained from Ni(OH)_2_ nanoparticles was centrifuged for the separation of particles from a solution. To produce the MWCNT/Ni(OH)_2_ composite as a modifier of the surface of the glassy carbon electrode (GCE) and a matrix for stabilization of the immune complex components of the immunosensor, 10 mg of MWCNTs solution was sonicated for 30 min to separate the MWCNTs from each other well. Next, Ni(OH)_2_ nanoparticles were slowly added to the MWCNTs solution under continuous stirring for 16 h at 60 °C. The obtained MWCNT/Ni(OH)_2_ nanocomposite was then cooled to room temperature centrifuged, washed several times, and placed in an oven at 60 °C for 1 h to eliminate all impurities.

### 2.5 Step-by-step modification of the GCE

**Fig. 1:**
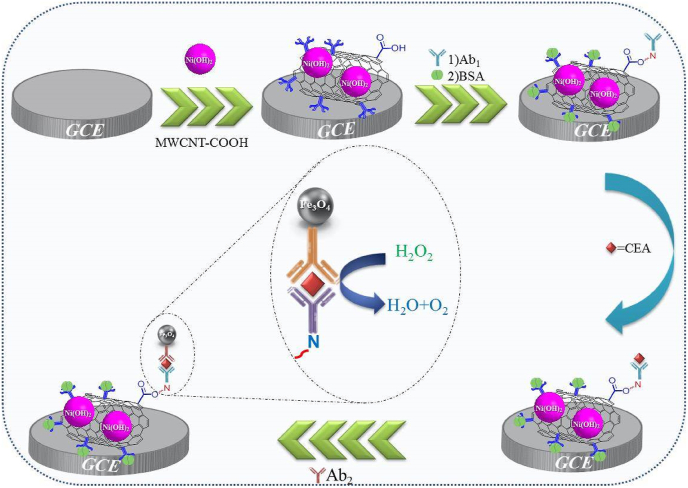
Schematic illustration of the designed CEA immunosensor.

For the initial modification of the GCE, first, its surface underwent a polishing process using a pad and alumina powder. To eliminate the excess material, the surface was washed with distilled water several times. The step-by-step assembly of the immunosensor is shown in the schematic of Fig. 1. In the next step, the GCE was immersed in the synthesized MWCNT/Ni(OH)_2_ nanocomposite solution for 4 h until the nanocomposite was completely absorbed in the surface. MWCNT/Ni(OH)_2_ composite was attached to the GCE surface through physical adsorption. The nanocomposites were uniformly distributed on the surface of the GCE due to the van der Waals forces between the Ni(OH)_2_ nanoparticles and the GCE surface. Next, the electrode was placed in a 60 °C oven for 30 min to completely dry the surface covered with nanocomposite. Then 10 μL of primary monoclonal Anti-CEA antibody (Ab_1_) with a concentration of 0.5 mg/mL was dropped onto the electrode with a sampler, and the electrode was left for 2 h until the antibody through its amine group interacted with the carboxyl groups of MWCNTs and stabilized on the electrode surface. To occupy non-specific binding sites on the electrode, it was immersed in 5 % BSA for 15 min, and then, to achieve the sandwich form of the immunosensor, the CEA antigen was incubated with the modified electrode for 75 min. Next, the Ab_2_s conjugated with Fe_3_O_4_ nanoparticles were added to react with CEA attached to the electrode surface and allowed to interact with CEA antigen for 60 min. The obtained immunosensor was kept at a temperature of 4 °C until use.

## 3 Results and discussion

### 3.1 Investigating the characteristics of MWCNT/Ni(OH)_2_ nanocomposite

**Fig. 2:**
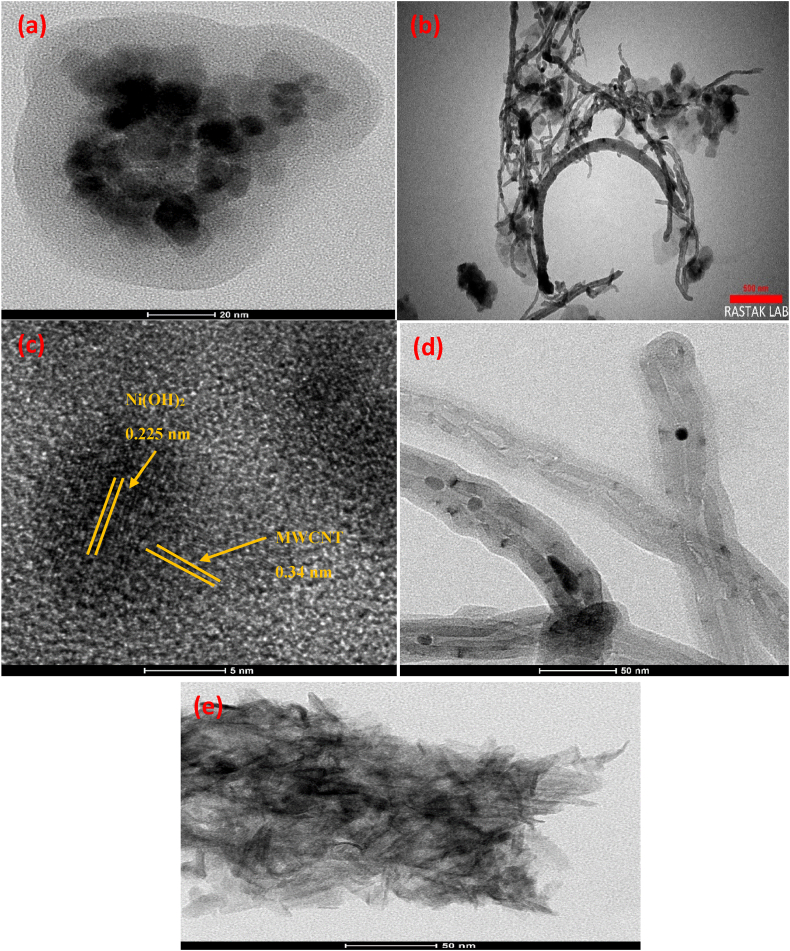
HRTEM images of (a) Fe_3_O_4_, (b and c) MWCNT/Ni(OH)_2_ nanocomposite, (d) MWCNT, and (e) Ni(OH)_2_.

The structure and morphology of Fe_3_O_4_ nanoparticles and synthesized MWCNT/Ni(OH)_2_ nanocomposite were investigated by an HRTEM [34]. Fe_3_O_4_ nanoparticles are shown in HRTEM images as in Fig. 2a. As shown in Fig. 2b, Ni(OH)_2_ nanoparticles were uniformly dispersed on MWCNTs. The nanocomposite has formed a large surface suitable for attaching immune components and completely covering the GCE. Ni(OH)_2_ nanoparticles had dimensions of about 40 nm. As shown in Fig. 2c, with a higher magnification image, the distance between planes is 0.34 nm for MWCNTs (d002), which corresponds to the distance between graphite layers [35]. The image also exhibits the fringes with a lattice distance of 0.225 nm, which matches the (101) plane of Ni(OH)_2_ [36].

**Fig. 3:**
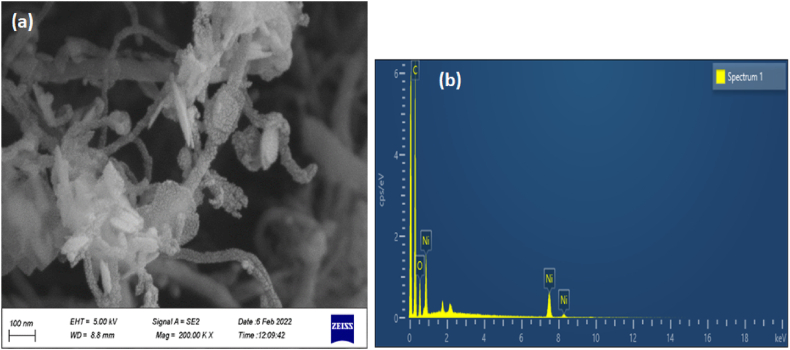
(a) FESEM image of MWCNT/Ni(OH)_2_ nanocomposite, (b) EDX spectrum of MWCNT/Ni(OH)_2_ nanocomposite.

One of the characteristics of metal nanoparticles is to prevent the agglomeration of MWCNTs [37]. FESEM images for MWCNT/Ni(OH)_2_ nanocomposite show the uniform distribution of Ni(OH)_2_ nanoparticles on MWCNTs (Fig. 3a). EDX analysis given in Fig. 3b for MWCNT/Ni(OH)_2_ nanocomposite reveals the existence of O, C, and Ni elements, confirming the presence of Ni(OH)_2_ along with MWCNTs.

**Fig. 4:**
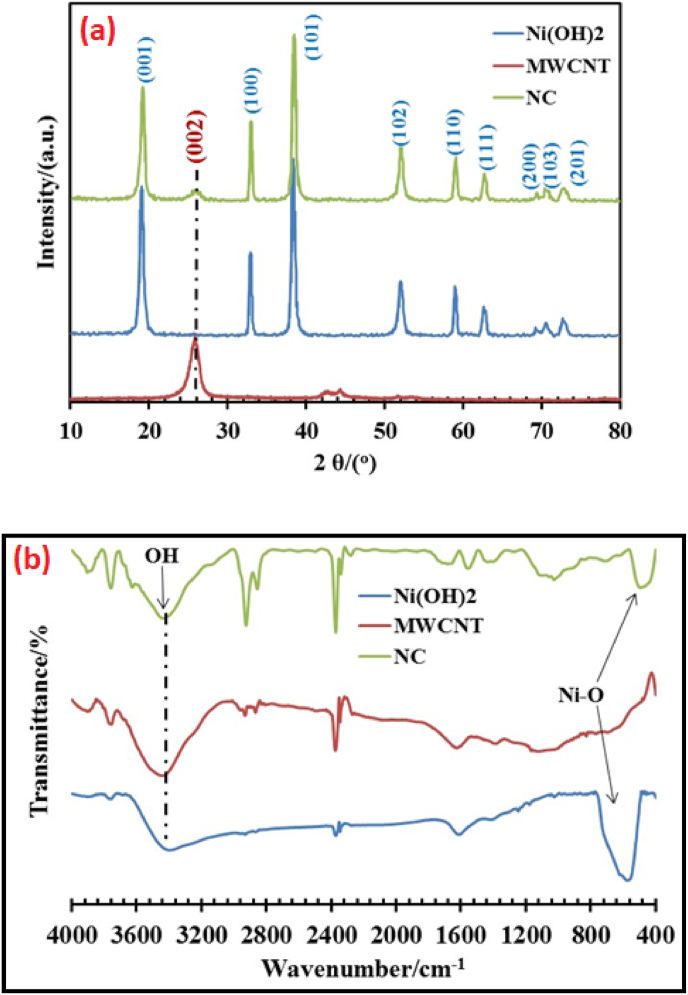
(a) XRD patterns and (b) FTIR spectra of Ni(OH)_2_ nanoparticles, MWCNT, and MWCNT/Ni(OH)_2_ nanocomposite.

Next, the properties of MWCNT/Ni(OH)_2_ nanocomposite, which has been fabricated using an innovative and straightforward technique, were investigated by comparing XRD and FTIR patterns for synthesized nanocomposite, Ni(OH)_2_ nanoparticles, and MWCNTs. In Fig. 4a, the XRD pattern for MWCNTs showed two peaks at 2θ around 26.5° and 44°, and a strong peak (002). In the graph related to Ni(OH)_2_ nanoparticles, there are clear peaks at 19.6°, 32.8°, 38.8°, 52°, and 59.4°, which correspond to the patterns of (001), (100), (011), (012), (110), and (111) planes, respectively. It was observed that by examining the previous patterns, the height of the peaks was reduced to a small amount in the composition of the nanocomposite, which indicated the successful combination of these materials with each other. The FTIR spectra for MWCNTs and Ni(OH)_2_ nanoparticles and synthesized nanocomposite matched the reference patterns (Fig. 4b). The stretching vibration of O–H bonds in the OH groups of Ni(OH)_2_ and MWCNT-COOH was seen as a wide peak within the region of 3200–3400 cm^−1^. The peak at about 1635 cm^−1^ was related to the bending vibration of H–O–H bonds in the hydroxyl groups of Ni(OH)_2_ nanoparticles. A peak at about 480 cm^−1^ corresponded to the bending vibration of Ni–OH bonds in the Ni(OH)_2_ structure. A peak at about 1600 cm^−1^ was attributed to the stretching vibration of C=C bonds in the structure of MWCNTs, and a peak at about 1400 cm^−1^ corresponded to the bending vibration of C–H bonds in MWCNTs. In the case of the FTIR spectrum of MWCNT/Ni(OH)_2_, any change in peak position or intensity was due to the interaction between the components of the nanocomposite.

### 3.2 Electrochemical characteristics of the developed immunosensor

**Fig. 5:**
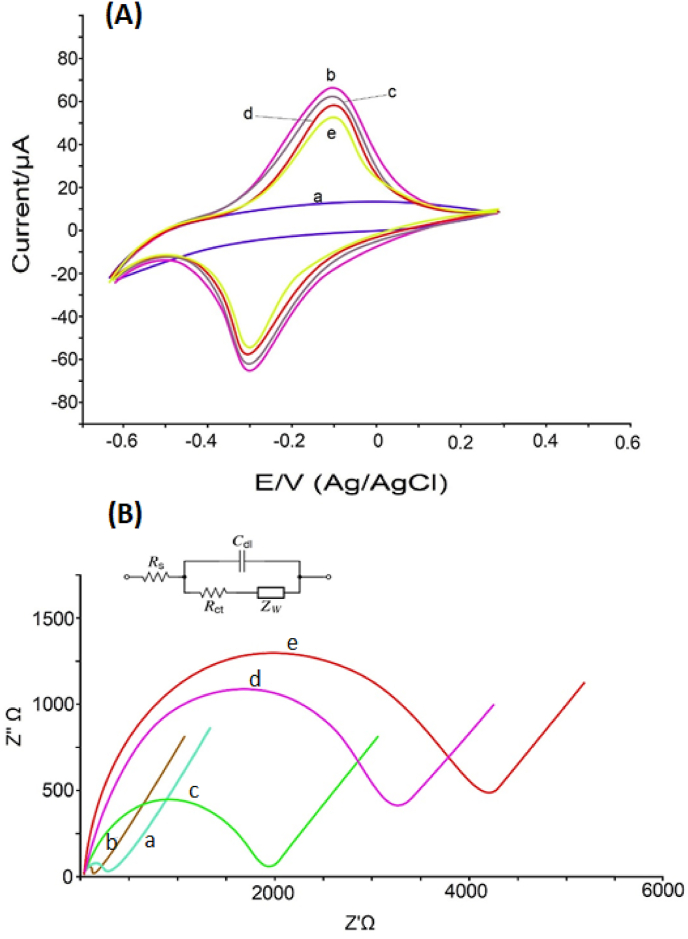
(A) CVs at scan rate 50 mV/s and (B) EIS obtained from: (a) bare GCE, (b) GCE/MWCNT-Ni(OH)_2_, (c) GCE/MWCNT-Ni(OH)_2_/Ab_1_, (d) GCE/MWCNT-Ni(OH)_2_/Ab_1_/CEA, (e) GCE/MWCNT-Ni(OH)_2_/Ab_1_/CEA/Ab_2_- Fe_3_O_4_, in 2.5 mM K[Fe(CN)_6_]^−3/−4^ with 0.1 M KCL.

To examine the electrochemical behavior of the designed immunosensor, the cyclic voltammetry (CV) employed for the GCE in a cell containing 2.5 mM K[Fe(CN)_6_]^−3/−4^ and 0.1 M KCl at various stages of surface modification [38] is presented in Fig. 5A. Redox peaks for bare GCE, GCE/MWCNT/Ni(OH)_2_, GCE/MWCNT-Ni(OH)_2_/Ab_1_, GCE/MWCNT-Ni(OH)_2_/Ab_1_/CEA, and GCE/MWCNT-Ni(OH)_2_/Ab_1_/CEA/Ab_2_ were obtained. In the CV related to the bare GCE, a significant peak did not appear due to the inability of the electrode to exchange electrons with the electrolyte. In the CV of the GCE modified by nanocomposite, the height of the peaks rose due to the faster exchange of electrons on the surface of the electrode. When the Ab_1_ was added to the electrode surface and reacted with the carboxyl groups on the MWCNTs surface, the CV measurements showed a decrease in current, which was due to the successful immobilization of Ab_1_ on the electrode surface. In the next step, when CEA was added to the immune complex, the current peak experienced another drop, which was caused by the existence of the insulating coating on the electrode surface. In the last step, the Ab_2_-Fe_3_O_4_ was added to the electrode surface, and the peak current was reduced again, which indicated the stabilization of the layer-by-layer surface-modifying components and immune materials on the electrode [39]. Also, in another method, the assembly process of electrode modifier materials in the manufacture of immunosensors was investigated by electrochemical impedance spectroscopy (EIS) [40]. For the designed immunosensor, the Nyquist diagrams obtained by this method consist of a linear part called the Warburg element that occurs at a low frequency associated with the process of diffusion and a semicircular part that is related to resistance (R_et_) at a high frequency [41]. According to Fig. 5B, GCE/MWCNT-Ni(OH)_2_ compared to bare GCE, which had a small resistance to electron transfer (R_et_ = 230Ω), is almost a straight line with a semicircular part with a much smaller diameter [42]. In the instance of GCE/MWCNT-Ni(OH)_2_/Ab_1_, the presence of the antibody insulating layer on the electrode increased the resistance dramatically (R_et_ = 1800Ω). In the next state of material assembly on the electrode, which was GCE/MWCNT-Ni(OH)_2_/Ab_1_/CEA, with the presence of more layer resistance to the electron exchange on the GCE, the value of R_et_ increased 3700Ω. In the last step, when the Ab_2_ was added to the immune components on the electrode and the structure of the immunosensor was completed in the form of GCE/MWCNT-Ni(OH)_2_/Ab_1_/CEA/Ab_2_, the electron transfer rate decreased again because of the resistance (R_et_ = 4400Ω). All these confirmed that the immunosensor was designed correctly.

### 3.3 Mechanism of electrochemical reactions

Fe_3_O_4_ with its peroxidase-like property can catalyze the H_2_O_2_ reduction reaction. The reason for using these nanoparticles conjugated with the Ab_2_ in the structure of the immunosensor is their higher stability in binding to the antibody and the ease of creating optimal pH and other conditions in the absence of natural peroxidase enzymes like HRP [43]. The catalytic reaction of H_2_O_2_ by Fe_3_O_4_ nanoparticles is given in equation 1.

$$H_{2}O_{2}+\text{Ni}{\left(\text{OH}\right)}_{2\left(\text{red}\right)}\overset{\text{Fe}304\left(\text{as}\;a\;\text{peroxidase}\right)}{\to }\text{Ni}{\left(\text{OH}\right)}_{2\left(\text{OX}\right)}+H_{2}O$$

Ni++(ox)+e–→Ni(red)+

### 3.4 Optimizing the immunosensor performance

**Fig. 6:**
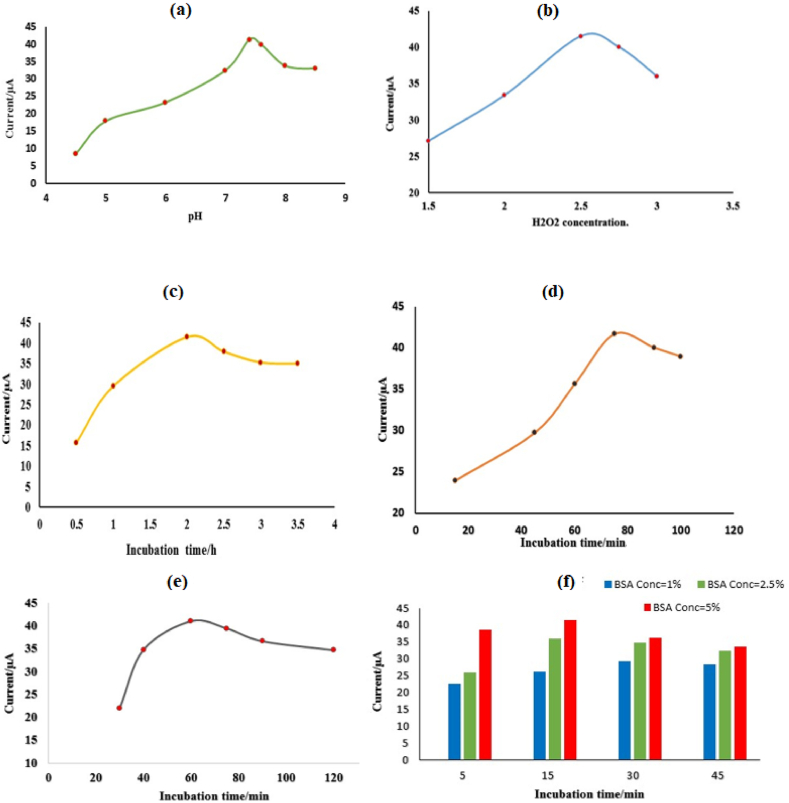
(a) Optimization of the pH, (b) Concentration of H_2_O_2,_ (c) Ab_1_ incubation time, (d) Interaction time of CEA and Ab_1_, (e) Incubation time of Ab_2_, and (f) Incubation time and concentration of BSA.

To achieve reliable results, the operating conditions of the presented immunosensor should be controlled, and in this regard, the pH, H_2_O_2_ concentration, and incubation time of the immune components were optimized [44]. To optimize the pH of the buffer, the performance of the immunosensor was examined in the range of 4.5–8.5, and a pH = 7.4 was determined as the optimal pH of the buffers (Fig. 6a). The H_2_O_2_ solution from 1.5 mM to 3 mM was used to measure the concentration of 2 ng/mL CEA, and the best diagnostic response was obtained at a concentration of 2.5 mM of H_2_O_2_ (Fig. 6b). Next, to better stabilize the components of the immune complex, the incubation time of the Ab_1_ with MWCNT/Ni(OH)_2_ was investigated as shown in Fig. 6c, and the highest current was obtained during the incubation time of 2 h for the measurement of 2 ng/mL CEA by differential pulse voltammetry (DPV) method. The incubation time of CEA with the Ab_1_ attached to the nanocomposite was investigated in the time range of 15–100 min, and the optimal time was 75 min (Fig. 6d). The time required for the maximum binding of the Ab_2_-Fe_3_O_4_ with the immune complex on the electrode surface was also optimized, and according to Fig. 6e, the maximum current value was recorded in 60 min. The concentration of BSA and incubation time of the modified electrode with it were optimized and the 5 % BSA solution was selected as the optimal concentration. The highest response for 2 ng/mL CEA detection was achieved in 15 min incubation time (Fig. 6f).

### 3.5 CEA detection with GCE/MWCNT-Ni(OH)_2_/Ab_1_/CEA/Ab_2_

**Fig. 7:**
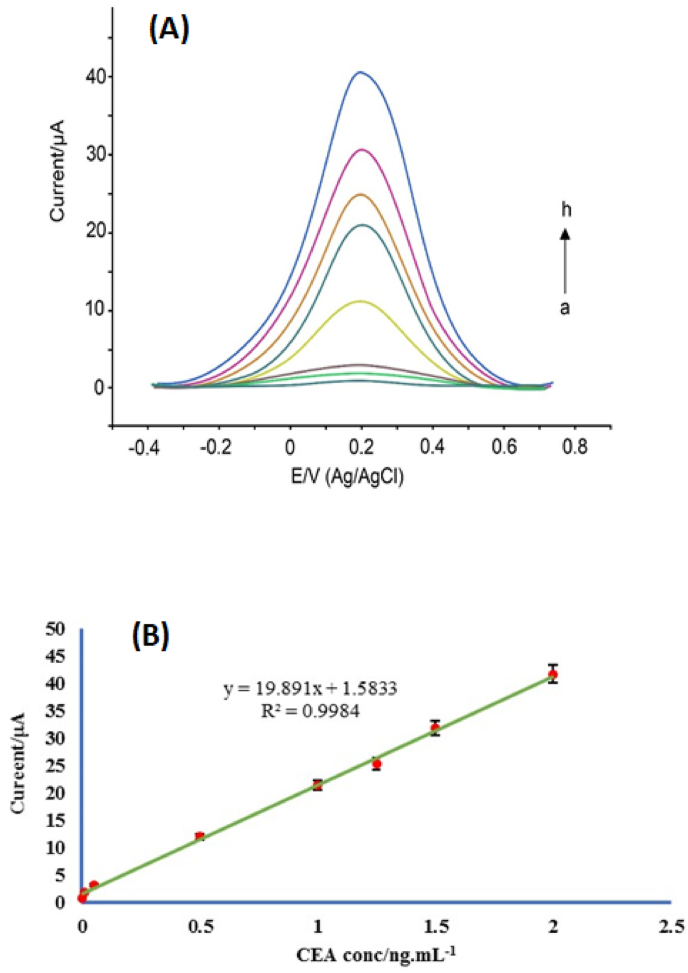
(A) DPV voltammograms of immunosensor following the different amounts of CEA (a to h): 0.0001 ng/mL, 0.01 ng/mL, 0.05 ng/mL, 0.5 ng/mL, 1 ng/mL, 1.25 ng/mL, 1.5 ng/mL and 2 ng/mL, (B) Calibration plot of current against various concentrations of CEA for the immunosensor under the optimization conditions. Error bar = RSD (n = 3).

CEA was detected by DPV technique [45]. All measurements were performed under optimal conditions. Fig. 7A shows the responses of the immunosensor with the successive addition of CEA antigen at different concentrations. When the measurements went to higher CEA concentrations in the presence of more amounts of tracer antibody (Ab_2_), the reduction current escalated due to the increase in the catalytic reaction between Fe_3_O_4_ conjugated to the Ab_2_ and H_2_O_2_. By plotting the calibration curve in Fig. 7B–a linear correlation was seen between the reduction currents and the antigen concentrations within the range of 0.0001 ng/mL to 2 ng/mL, which was a linear regression equation (y = 19.891x + 1.5833) with a correlation coefficient of R^2^ = 0.9984. The LOD value for the CEA was calculated to be 0.076 pg/mL. Compared to the similar immunosensors previously reported in Table 1 [46–54], the modified GCE based on MWCNT/Ni(OH)_2_ showed a better response for the determination of CEA in the form of a new electrochemical immunosensor. This can be related to MWCNTs in combination with Ni(OH)_2_ which can be extensive electrical pathways, leading to the increase of electrochemical response.

**Table 1:** Comparison of different CEA immunosensors.

Material on electrode	Linear range	LOD	Ref.
graphene/Cu	1.0–25.0 ng/mL	0.23 ng/mL	[46]
3D-rGOMWCNTs/Ag–Au NPs	0.0001–80.0 ng/mL	0.003 ng/mL	[47]
[Ag–Ag_2_O]/SiO_2_	0.5–160 ng/mL	0.14 ng/mL	[48]
ITO/PANI/PPy-Ag	0.001–100 ng/mL	0.4 pg/mL	[49]
rGO/MoS_2_@PANI	0.001–80.0 ng/mL	0.3 pg/mL	[50]
AuNPs-PAN@CNTs	0.002–80 ng/mL	8 pg/mL	[51]
AgNC@Apt@UiO-66	0.01–10 ng/mL	300 pg/mL	[52]
AuNPs-Thi -MoS_2_	0.005–10 ng/mL	0.52 pg/mL	[53]
CNSs@Au NPs	0.002–80 ng/mL	3 pg/mL	[54]
MWCNT/Ni(OH)_2_	0.005–4 ng/mL	0.076 pg/mL	**Present work**

### 3.6 Reproducibility, stability, and selectivity

**Fig. 8:**
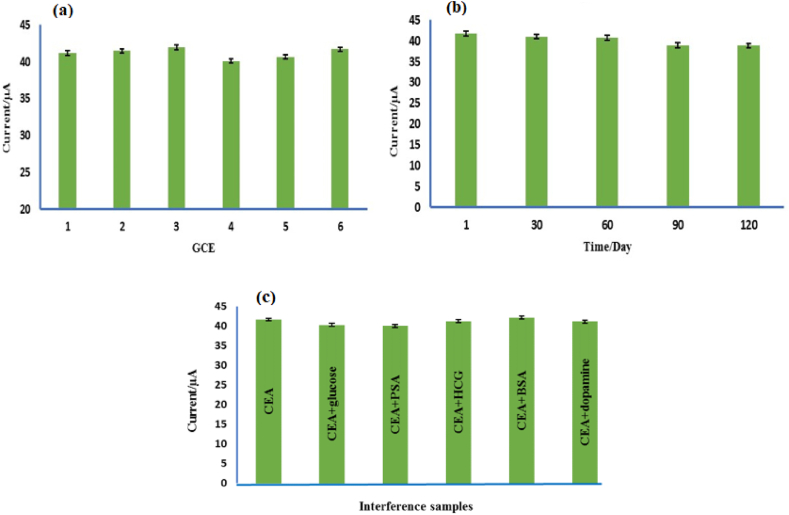
The results for (a) reproducibility of the immunosensor (b) stability of the modified electrode, and (c) interfering agents affecting the immunosensor.

To study the reproducibility of the developed immunosensor, 2 ng/mL of CEA was measured separately by six electrodes that were prepared under identical experimental conditions. Very close results were obtained in the current measurements with the electrodes prepared so that the current obtained from the measurement of 2 ng/mL of CEA for electrodes 1 to 6 respectively were 41.197 μA, 41.51 μA, 41.984 μA, 40.12 μA, 40.679 μA, and 41.716 μA. The findings showed a relative standard deviation (RSD) of 1.5 %, which proves that this immunosensor design method was highly reproducible (Fig. 8a). It is very important to maintain the accurate measurement performance of an immunosensor, so the immunosensor must have good stability. With the prepared immunosensor, 2 ng/mL of CEA was measured on the first day by the DPV method, then the immunosensor was stored at 4 °C and used again to measure the same amount of CEA every 30 days. After 120 days, 93.19 % of its initial response was maintained, which showed the stability of the measurements in the long term (Fig. 8b). Also, the effect of interference was examined by measuring 2 ng/mL of CEA antigen together with samples containing 100 ng/mL of other substances that can be present in blood serum samples, such as glucose, PSA, HCG, BSA, and dopamine (The results of the currents measured by the DPV method for 2 ng/mL CEA alone and in the presence of interfering substances were 41.7 μA, 40.313 μA, 40.01 μA, 41.24 μA, 42.183 μA, and 41.11 μA, respectively). The results in Fig. 8c indicate that the RSD of the responses was 1.8 %. This implies that the presence of these interfering substances did not have an impact on the measurement of CEA antigen and the immunosensor made by the new method provides excellent selectivity and reliable results.

### 3.7 Real samples examine

To assess the actual ability of the immunosensor based on GCE/MWCNT-Ni(OH)_2_, the measurement of CEA antigen in different blood serum samples prepared from Farabi Laboratory in Ardabil, Iran, was investigated. This project has been approved by regional ethics committee, Ardabil University of Medical Sciences. Antigen quantification was examined by the standard addition method. The results for increasing the spiked amounts to an initial amount of 1 ng/mL of CEA were obtained according to Table 2, and the RSD ranged from 1.04 % to 1.13 % and the recovery values for sample determination were 99 %, 101.9 %, and 106 %. Hence, the findings of this study showed that the developed immunosensor can be practically used for the determination of CEA in clinical settings. To examine the measurement accuracy of the presented method, a comparison of the measurement of CEA values by the immunosensor with the ELISA method was made, and the difference in the obtained results was very close (Table 3).

**Table 2:** CEA concentration in blood serum samples detected by the proposed immunosensor.

CEA sample (ng/mL)	Spiked (ng/mL)	Measured concentration (ng/mL)	Average (ng/mL)	RSD% (n = 5)	Recovery% (n = 5)
	0.05	1.076,1.04,1.0502,1.05, 1.05	1.053	1.13	106.6
1	0.25	1.27, 1.238, 1.245, 1.261, 1.253	1.254	1.07	101.9
	0.75	1.723, 1.721, 1.749, 1.750, 1.769	1.725	1.04	99

**Table 3:** Comparison of the CEA measurement with two methods.

Sample	Immunosensor method (ng/mL)	ELISA method (ng/mL)
1	1.15 ± 0.12	1.2
2	1.66 ± 0.25	1.6
3	0.53 ± 0.02	0.5
4	0.21 ± 0.05	0.23

## 4 Conclusion

We presented a new strategy for the development of an electrochemical CEA immunoassay based on MWCNT/Ni(OH)_2_ nanocomposite. Also, Fe_3_O_4_ nanoparticles were used instead of the HRP enzyme conjugated with the antibody, which contributed to the high stability of the immunosensor. Synthesis of the nanocomposite was carried out with a simple, fast, and low-cost operating method. The binding of Ab_1_ to a magnetic nanocomposite was realized by the covalent bond between the amine groups of antibody molecules and the COOH groups of MWCNTs. The use of MWCNT/Ni(OH)_2_ nanocomposite along with Fe_3_O_4_ nanoparticles instead of HRP enzyme can be an advance of the proposed method, which was also confirmed by the long stability of the immunosensor. The results showed that the fabricated immunosensor had advantages such as a wide linear detection range, low detection limit, and acceptable reproducibility. The proposed electrochemical immunosensor has good potential to be incorporated as a platform for commercial and clinical purposes.

## CRediT authorship contribution statement

**Ali Shamsazar:** Methodology, Investigation, Formal analysis, Data curation. **Mahsa Soheili Moghaddam:** Investigation, Formal analysis, Conceptualization. **Asadollah Asadi:** Visualization, Software, Conceptualization. **Majid Mahdavi:** Validation, Supervision, Project administration, Investigation, Conceptualization.

## Declaration of competing interest

The authors declare that they have no known competing financial interests or personal relationships that could have appeared to influence the work reported in this paper.
